# Socio-Economic Determinants of Health Literacy in High School Students: A Cross-Sectional Study

**DOI:** 10.3390/ijerph182212231

**Published:** 2021-11-21

**Authors:** Mariusz Duplaga, Marcin Grysztar

**Affiliations:** Department of Health Promotion and e-Health, Institute of Public Health, Faculty of Health Sciences, Jagiellonian University Medical College, 31-066 Kraków, Poland; marcin.grysztar@uj.edu.pl

**Keywords:** high school students, adolescents, health literacy, health literacy sub-indices, European Health Literacy Survey Questionnaire, paper-and-pencil interviewing, logistic regression, linear regression

## Abstract

Today, a person’s health literacy (HL) is perceived as a critical determinant for their health. It is well known that limited health literacy is associated with harmful health behaviours, the lower use of preventive services, problems in making use of healthcare resources and poor skills for the self-management of chronic disorders. This study analysed the level and factors associated with HL in high school students in southern Poland. The study is based on the data from a paper-and-pencil survey of high schools students in the Malopolska Voivodshop. The students were selected using a two-stage cluster random sampling procedure. The level of HL was assessed with the European Health Literacy Survey Questionnaire, consisting of 47 items. Univariate logistic and linear regression models were applied to analyse the association between general HL score and the main domain-specific indices. The respondents whose parents had attended University and came from more affluent families were less likely to demonstrate limited HL. There was a significant positive relationship between the self-assessment of the economic situation, size of accommodation, the level of expenditure on a mobile phone and receiving external support, the size of the book collection at home, and domain-specific HL sub-indices. In conclusion, among socio-demographic factors, the parents’ education is the only consistent predictor of the level of HL in high school students. Furthermore, most variables reflecting the economic status of the respondent’s family are significantly associated with the general HL score and its sub-indices.

## 1. Introduction

The term health literacy (HL) is used in relation to the cognitive and social skills which have an impact on the motivation and ability of people to access, understand and use information in order to promote and maintain good health [1]. The evidence from the available research shows that HL may be an important determinant of health behaviours, the use of preventive services, the way that a person interacts with the healthcare system and the self-management of their long-term conditions [2,3,4,5,6]. The importance of HL in modern health promotion was recognised in the Global Health Promotion Conference in Shanghai held in 2016 when it was declared as being a critical determinant of health [7].

It was shown that the level of HL, depending on the study sample, may be associated with sex, age, level of education, economic and social status, and professional background [3,6,8,9,10,11,12,13].

There are three types of instruments used for the assessment of HL: relying on objective measures (performance-based tests); those based on subjective, self-reported measures, or applying mixed-method measurements [14,15]. The instruments may be designed to assess general HL or may be focused on specific domains, e.g., diabetes, oral health, etc. [16]. According to Liu et al. [17], all 11 tools used for the measurement of the general population’s HL of were based on multidimensional concepts until 2018. Furthermore, they also distinguished three types of instruments, but their classification focused on health-related fields (e.g., mental health or public health) and health-related abilities, such as accessing, appraising and applying health information.

Some authors have indicated that the HL of children and adolescents has not been as frequently researched as that of the adult population, [15,18]. In 2014, Perry identified only ten studies that had focused on the HL of adolescents [18]. In the review published four years later, Okan et al. [15] identified 15 instruments used in studies carried out on children and adolescents. Apart from dedicated tools designed for children or adolescents, such as the Rapid Estimate of Adult Literacy in Medicine–Teen (REALM-teen) [19], or the Health Literacy Questionnaire for Children [20], the general HL tools, e.g., the European Health Literacy Survey Questionnaire (HLS-EU-Q47) were used [21] or the Newest Vital Sign (NVS) [22]. Bröder et al. developed a systematic review of the definitions and models of HL in children and youths [23]. They identified as many as 12 definitions and 21 models specifically developed for children and young people. They concluded that in the literature, HL in these groups is usually described as a set of dimensions that can be interpreted as clusters of related abilities, skills, commitments and knowledge, which facilitate the competent handling of health information and making effective health-promoting decisions. It seems that the definitions of HL proposed in the context of children and young people sound similar to iterations of the definitions used for general HL.

The determinants of the HL of high school students has been assessed in several studies. Wu et al. found that among 10th grade students in a secondary school in Vancouver, the HL score measured with a newly developed tool was significantly associated with gender, speaking English as a second language, immigration to Canada at a later age and frequent absence from school [24]. Röthlin et al. analysed the HL of 15-year-old Austrian youths. In this group, HL was significantly associated only with the level of education achieved by parents, the family’s economic status, and the location of residence [21]. The level of HL of Iranian high school female students measured with the Health Literacy Measure for Adolescents (HELMA) tool was associated with variables related to educational level, but not with other demographic variables [25].

There were also several studies analysing the association between HL and health-related outcomes. A significant relationship between low HL and the prevalence of overweight and obese 12–16 years old pupils in China was described by Lam and Yang [26]. Park et al. reported that in 9th grade students from an urban school district, a lower HL was associated with lower self-assessed health, unhealthy diet, greater weight and higher involvement in risky behaviours [27]. A systematic review focused on HL in adolescents, and young adults showed that smoking and obesity was related to lower HL [28]. Among subjects with a chronic disease, the findings on the relationship between HL and adherence to routines medication were not unequivocal [28]. The association of the HL and adolescents’ health behaviour was confirmed in a systematic review prepared by Fleary et al. in 2018 [29].

In this study, we have analysed the level and socio-economic determinants of HL measured with the 47-item version of the questionnaire developed by Sorensen et al. within the European Health Literacy Survey Project (HLS-EU-Q47) [6]. Furthermore, the factors associated with the main HL sub-indices derived from the questionnaire responses have also been assessed.

## 2. Materials and Methods

### 2.1. Survey

The paper-and-pencil survey was performed on students attending high schools located in the Malopolska Voivodship in southern Poland. Two-stage cluster random sampling was applied. In the first stage, 20 schools were randomly selected from the list provided by the local Board of Education. Of these schools, the directors of nine agreed to participate in the study. In the second stage, a sample of classes from each school was randomly selected in order to obtain a balanced representation of successive years and thematic profiles. In general, the study team attempted to involve at least five classes from a school, but not more than 10 in a very large school. All students in the selected classes were invited to participate in the survey. The criteria for inclusion in the study were: the consent to participate in the survey; and in case of minors, the consent of a parent or legal guardian and being aged 15–20.

The questionnaire used for the study included a Polish version of the 47-item tool developed in the European Health Literacy Survey Project (HLS-EU-Q47) [6], an 18-item version of the Multidimensional Health Locus of Control Scale [30], a set of questions asking about health behaviours and items focusing on socio-demographic and economic circumstances of the respondents. In total, the questionnaire consisted of 130 items.

The study received the consent of the Bioethical Committee of Jagiellonian University (decision from 25 September 2014, No KBET/193/B/2014). Before the study commenced, the students were informed about the aims of the study and their agreement to participate was obtained. In the case of minors, parental consent was required. The survey was anonymous, and students could resign from the study at any moment.

### 2.2. General Health Literacy Index and Sub-Indices

The HL of respondents was measured using the standard version of the European Health Literacy Survey Questionnaire consisting of 47 items (HLS-EU-Q16). The respondent completing the questionnaire could select one of five options ranging from “very easy” to “very difficult” and “difficult to say/not applicable”. The general HL score and the seven main HL sub-indices were calculated according to the guidelines provided by the HLS-EU project team [31]. When a respondent could not assign an option to an item, or did not consider it was applicable, that item was treated as a missing value. The responses from “very easy” to “very difficult” were scored from 4 to 1. The HL score was calculated using the formula: (mean—1) × (50/3), when missing values were less than 20%. Sub-indices for the domains related to the handling of health information: accessing (AI-HL), understanding (UI-HL), appraising (ApI-HL) and using (Us-HL), and for three areas of health services: healthcare (HC-HL), disease prevention (DP-HL) and health promotion (HP-HL) were calculated using the same formula after including only the responses of relevant items. Then, the general HL index and its sub-indices were transformed to a unified metric assuming a minimum of 0 for the least possible and a maximum of 50 for the best possible score [6].

The assessment of the general HL and domain-specific sub-indices assessment showed an acceptable, or very good level, of reliability of general index and sub-indices. The values of Cronbach-α and Guttman split-half coefficients for all HL indices are presented in Table 1.

Based on the general HL score, four HL levels were distinguished in accordance with the recommendations of Sørenson et al. [6]: inadequate—from 0 to 25; problematic—from >25 to 33; sufficient—from >33 to 42 and excellent from >42 to 50.

### 2.3. Statistical Analysis

The IBM SPSS Statistics v.26 software package was used for the statistical analysis (IBM Corp. Armonk, NY, USA). Descriptive statistics were calculated for categorical variables, included the absolute and relative frequencies. Numerical continuous variables were described with the mean and standard deviation (SD).

Univariate logistic regression models were developed for the assessment of the predictors of the prevalence of limited literacy. The dichotomous dependent variable was established based on the general HL transformed to categorical variables assuming four levels. Then, inadequate and problematic categories were collapsed into a limited HL category and sufficient and excellent categories into a non-limited HL category. For each model, the Hosmer and Lemeshow chi2 test and the Nagelkerke R square were obtained. The odds ratio (OR), 95% confidence interval (95% CI) and *p*-value, were presented for independent variables. These included gender place of residence, parents’ education level, parent’s marital status, the number of members in the household, the year of study and type of the school, the size of accommodation, receiving support from external sources, self-assessed economic status, monthly expenditure on a mobile phone, the size of the book collection at home and the number of hours per week spent on the Internet (please see Supplementary File S1). The independent variables having significant relationships confirmed by the univariate regression models were included in the multivariate logistic regression model. The relevant adjusted values of OR, 95% CI and *p*, are reported for the model.

The associations between the main seven HL sub-indices and independent variables were analysed with univariate linear regression models. The results of linear regression were reported as unstandardised regression coefficients (B), standard errors (SE), standardised regression coefficients (β), 95% confidence intervals (95% CI), and *p* values were provided for the independent variables. The level of *p*-value significance was assumed at the level < 0.05. The *p* values were reported to three decimal places.

## 3. Results

### 3.1. Characteristics of the Study Group

The size of the study group, after quality control of the returned questionnaires, was 2223. The response rate was 95.4%. The mean age of the respondent was 17.01 years (SD = 0.97) and 66.29% (*n* = 1457) of the respondents were female. Of all respondents, 82.31% (*n* = 1829) studied at high schools providing general education (lyceums). Detailed characteristics of the participants are shown in Table 2.

### 3.2. Health Literacy

**Figure 1:**
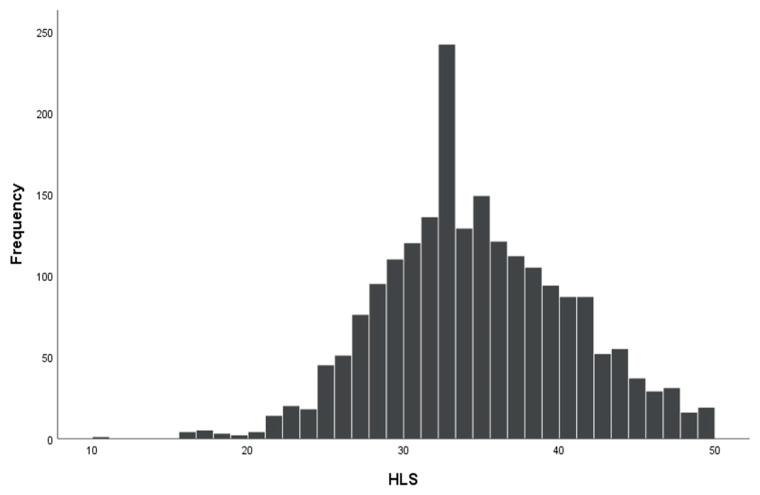
The distribution of HL score values in the study sample.

The mean HL score was 34.76 (SD = 6.14). The sub-indices reflecting handling the information exhibited the following mean values: AI-HL—34, 35 (SD = 6.71), UI-HL—36.22 (SD = 6.82), ApI-HL—33.17 (SD = 7.40), and UsI-HL—35.62 (SD = 7.00). Furthermore, HC-HL was 35.35 (SD = 6.15), DP-HL 33.99 (SD = 7.28), and HP-HL 34.56 (SD = 7.40). Inadequate HL was possessed by 4.61% (*n* = 96) of the respondents, problematic by 36.79% (*n* = 766), sufficient 45.24% (*n* = 942) and excellent by HL 13.35% (*n* = 278) (Table 1). For 6.34% (*n* = 141) respondents, the general HL score could not be calculated because of the missing values exceeding 80% of the responses to the items in HLS-EU-Q47. The values of general HL score typically showed a left-skewed, distribution (Figure 1) as reported for other populations [6].

The univariate logistic regression models were developed for assessing the prevalence of limited HL (Table 3). They showed a significant relationship with the levels of education of both parents and with several independent variables related to the economic situation of the respondents and their families. If the mother of the respondent was a University graduate, the odds of limited HL was 27% lower than in the case of a mother with only primary or vocational education (OR, 95% CI: 0.73, 058–0.920). In the case of a father with a University education, the odds of limited HL was 20% lower than for respondents whose father’s level of education was lower than secondary (OR, 95% CI: 0.80, 0.64–0.997). Respondents from a household with an inhabitable area of 70 m^2^ to below 90 m^2^ and at least 90 m^2^ were 42% and 24% less likely to have limited HL than those living in accommodation with an area less than 50 m^2^ (OR, 95% CI: 0.58, 0.39–0.85, and 0.74, 0.54–0.99, respectively). Students able to spend more than 50 Polish zlotys (PLN) per month for their mobile phones also showed a significantly lower probability of having limited HL than those spending smaller amounts (OR, 95% CI: 0.62, 0.40–0.96). Respondents from households receiving external support also had a lower chance of having limited HL than those in households lacking such support (OR, 95% CI: 0.81, 0.68–0.97). Simultaneously, respondents who self-assessed the economic situation of their families as very good, or good, had higher HL than those assessing their situation less favourably (OR, 95% CI: 0.37, 0.28–0.49, and 0.59, 0.46–0.77, respectively). Finally, the students from homes with the largest collections of books had 34% less likelihood of having limited HL than those from homes with the smallest collections (OR, 95% CI: 0.66, 0.45–0.98).

The prevalence of limited HL was not related to gender, class and type of school, the marital status of parents, the number of people in the household and the place of the residence. Additionally, it was unexpected that the level of HL was not associated with the intensity of Internet use.

After developing a multivariate logistic regression model with significant predictors revealed in the univariate models, only the parents’ level of education, the self-assessed economic situation, and the inhabitable size of the accommodation maintained significant relationships with the prevalence of limited HL (Table 3). The likelihood of limited HL was lower in respondents whose mothers had University education in comparison to those whose mothers had lower than secondary education (OR, 95% CI: 0.74, 0.55–0.98), whose fathers had secondary education than those whose father had lower than secondary education (OR, 95% CI: 1.32, 1.04–1.67), in respondents that assessed their economic situation as good in comparison to those assessing it as worse than good (OR, 95% CI: 0.65, 0.49–0.86), and finally, in respondents living in households with the size of accommodation from 70 m^2^ to below 90 m^2^ than those living in households with the area below 50 m^2^ (OR, 95% CI: 0.66, 0.43–0.998).

### 3.3. Predictors of Sub-Indices of Health Literacy

The education level achieved by the mother was significantly associated with all main HL sub-indices analysed for the paper. Respondents having mothers possessing University level education consistently had higher sub-indices than those with mothers who had reached only a secondary level of education (Table 4, Table 5 and Table 6). University education possessed by fathers was associated with students’ higher HL sub-indices than by those whose fathers had a secondary education, but only for AI-HL, UI-HL and DP-HL. Gender was a significant predictor for AI-HL and ApI-HL (Table 4 and Table 5); these sub-indices were significantly higher in males than females. All the identified associations were not high, and their effect on dependent variables did not exceed 10%.

Interestingly, the respondents studying in the III or IV class showed lower HP-HL (B (SE), *p*: −0.93 (−0.06), 0.017). Furthermore, students at the high schools providing vocational training had a lower UI-HL sub-index than students at the schools providing general education. The HL sub-indices were not significantly associated with the marital status of their parents, the number of people in the household or the place of residence.

Of the variables reflecting the economic status of the respondent’s family, significant association with HL sub-indices was found for the receipt of external support and the self-assessment of their economic situation. Reporting that the family received external support, financial or material, was associated with higher levels of all the sub-indices considered. However, the respondents assessing their economic situation as worse than good had lower HL sub-indices, apart from that related to healthcare, than those considering their situation as good. Furthermore, respondents who self-assessed their economic situation as ‘very good’ showed higher HL sub-indices for all seven areas compared to those assessing their situation as ‘good’ (Table 4, Table 5 and Table 6).

The largest inhabitable areas of the accommodation were significantly associated with higher sub-indices for accessing and using health information and the sub-index for healthcare compared to the referential category of inhabitable area. The respondents spending the most on their mobile phones showed higher HL sub-indices for accessing and appraising health information and the sub-index for healthcare than those whose spending on their mobile phone fell into a lower category.

Finally, all sub-indices were higher for the study participants in homes with the largest collections of books than those living in homes with smaller collections of books. The intensity of weekly Internet use was not statistically associated with the HL sub-indices.

## 4. Discussion

HL is perceived as a critical determinant of health [7]. Previous studies carried out among various populations have shown that there is a significant relationship between HL and many health-related outcomes, including the utilisation of health services, the ability to communicate with health care providers, and health behaviours [4]. A significant association between HL and health behaviours has also been reported for adolescents [26,27,28,29,32]. For example, Reid et al. observed that middle school students with lower HL showed a lower frequency of health-promoting behaviours, including physical activity [32]. The role of HL in shaping youth health behaviours has become particularly important during the COVID-19 pandemic when many young people have remained in their homes for prolonged periods. It seems that maintaining a moderate level of physical activity and nutritional habits may have a preventative effect against the prevalence of insomnia, depression, and anxiety symptoms in adolescents during the COVID-19 pandemic [33,34]. However, many studies have reported diminished physical activity in this population during the COVID-19 pandemic [35,36,37,38]. According to Sekulic et al., students who obtain higher grades at school are more aware of the health benefits related to physical activity [39]. Achievements at school that usually predict higher HL may be significantly associated with physical activity levels during lockdown related to the COVID-19 pandemic [39]. Unfortunately, according to the scoping review prepared by Rossi et al., none of the studies on the determinants of physical activity level during the COVID-19 pandemic have addressed the role of HL [38].

In 2008, Manganello [40] proposed a framework for studying adolescent HL based on a report from the US Institute of Medicine [41] and Bronfenbrenner’s ecological model [42]. The framework reflects the influence of individual characteristics that adolescents may have on HL, which then determines health outcomes. Apart from individual traits, the role of family and peer influences, as well as systems (education, healthcare, mass media) was incorporated into the framework. Both clusters of factors can influence individual traits, the level of health literacy, and health outcomes. The individual traits addressed in the framework included sociodemographic variables such as age, race, gender, education, culture, social and cognitive skills, physical abilities, and media use. The health outcomes covered such aspects as health behaviours, health costs and health service use.

The available studies reported a significant relationship between HL and many potential determinants addressed in Manganello’s framework, including gender [24], immigration status [24], self-efficacy [43], parents’ level of education [21], family economic status [31], place of residence [21], school environment [43], and performance at school, e.g., achievement or absence [24].

Bröder et al. proposed a 6-D model of HL in childhood and youth [44]. Overall, the dimensions of HL distinguished in this model can be compared to the factors included in Manganello’s framework [40]. However, the model more specifically emphasizes the sensitive areas of HL development. These authors underline the influence of social context for understanding key concepts such as ‘health” or ‘disease”. Furthermore, they point out that some health risks and diseases may be age- or development-specific. Demographic patterns are considered in relation to the resulting social and health inequalities. The 6-D model of HL also covers such dimensions as developmental change and the socialisation process, dependency within a power structure and inter-generational relationships, democratic citizenship and active participation, and finally, the digitization of the world of growing up. A detailed discussion of all these dimensions would exceed the scope of this paper. Therefore, we focus on the factors or dimensions addressed in Manganello’s and the 6-D model that have been operationalised in our study.

We have focused mainly on factors belonging to individual traits, family influences, and education system features. Furthermore, we have attempted to show what factors are associated not only with limited HL but also with subscores originating from the HLS-EU-Q47 tool. The prevalence of limited HL in the sample of Polish high school students exceeds 40% and, of the socio-demographic variables, HL is significantly associated only with their parent’s level of education. However, significant relationships can be seen for variables reflecting the economic status of the respondents and their families. The respondents from families receiving external support, those self-assessing their economic situation more favourably, spending the most on their mobile phones, and living in homes with the highest inhabitable area are 20–60% less likely to have limited HL. For other analysed potential predictors, only the number of books at home was significantly related to the likelihood of limited HL, but the intensity of Internet use was irrelevant. The HL sub-indices related to handling heath information and activities targeting health consistently showed significant relationships with the mother’s education level, the number of books at home and variables reflecting the economic status of the respondent, including the self-assessed economic situation and receiving external support. However, all these effects were limited and not greater than 10%. It is not clear why the better self-assessment of economic situation and living in a family receiving some form of external support were associated with higher levels of HL sub-indices.

The results of this study are in line with the findings of Röthlin et al. for 15-year-old youths in Austria [21]. In this group, they found that HL was associated with the parents’ education level, the level of affluence of the family, and the region of residence. Having at least one parent with a University education was related to a reduced likelihood of limited HL. Furthermore, those from families in the lowest category of affluence, measured with the Family Affluence Scale, were less likely to have sufficient or excellent HL than those in the higher categories of the FAS. There was also a significant relationship between HL and the region of the country. HL level was not associated with gender, the type of school attended, place of residence, level of education or the structure of the family.

The study undertaken by Park et al. [27] on a group of 250 adolescents of mean age 14 years entering, or in the 9th grade, of an urban school district in the USA showed that their HL measured with REALM and NVS scores were negatively correlated with age and positively with being of a white race. In our study, the school class, used as a measure of the age of the respondents, was not a significant predictor in the developed regression models for the total HL score, or for the sub-indices, apart from that for health promotion. In the latter case, attending the III or IV classes was associated with a lower sub-index.

The survey carried out by Dehghankar et al. on a group of 372 female Iranian high school students showed no significant relationships between HL and demographic variables apart from the father’s level of education and the class attended [25]. Respondents whose fathers had attained an intermediate level of education were more likely to show higher HL than those whose fathers had a lower level of education. In addition the survey showed that those who attended 12th class possessed higher HL than those in the 10th class.

Wu et al. [24] developed a tool and conducted the measurements of HL on 275, mainly 10th grade high schools students in Vancouver, Canada. They analysed HL sub-indices for domains related to understanding and evaluating health information and the overall scores. All three scores were lower for males than for females, in students whose parents had lower levels of education levels, in those who moved to Canada at an older age, and in those who achieved lower results at school. Contrary to our findings, the time spent on electronic activities was significantly positively associated with the domain and overall score. For the Polish students, family affluence was a consistent predictor of higher general and domain HL sub-indices. In the Canadian sample, it was positively associated only with the HL domain related to evaluating health information.

A systematic review carried out by Fleary et al. in 2018 [29] on the association between adolescent HL and health behaviours reported the results of the analysis between demographic variables and HL. A significant association was found between the age or the grade level, as a proxy for age, and the level of HL. Furthermore, of nine studies assessing the relationship between gender and HL one study reported higher HL for males and in three studies, for females. In five studies in the USA, three found lower HL in Black or Hispanic respondents than in White respondents. Three of the five studies found a significant relationship between lower family income or its proxys and lower HL. Finally, in five of six studies, a positive relationship was described between parents’ level of education and adolescents’ level of HL.

To sum up, the findings from our analysis are principally in line with the observations from earlier studies involving adolescents from various countries. However, of the demographic factors, only the mother’s level of education was a consistent predictor of higher general and domain-specific HL indices. The father’s level of education was a significant predictor of sufficient HL in the model developed for overall HL score and in three of the seven HL sub-indices. The variables reflecting family affluence showed a significant relationship with general and domain-specific HL scores. Higher scores for the respondents self-assessment of their economic situation, a more spacious accommodation and greater expenditure on a mobile phone were associated with higher HL. Of the other analysed factors, the size of the book collection at home was consistently associated with general and domain-specific HL sub-indices.

Unexpectedly, the time spent on the Internet was not associated with the level of HL. When proposing her framework of adolescence HL, Manganello pointed out that adolescents are particularly active in mass media [40]. Broder et al. included the digitalisation of growing up as one of six fundamental dimension of HL in childhood and youth [44]. Today, it is obvious that digital media play an important role in the process of growing up. Therefore, some authors use the term ‘digital natives’ in relation to younger generations as they seem immersed in the digital world and are able to use diversified tools to access information and communication with others [45]. Although the relationship between digital HL and the intensity of Internet use has been addressed in many studies, evidence regarding the role of the Internet in HL development is not very extensive. One could expect that more time on the Internet should be related to a higher level of HL in this group, but this was not the case among Polish high school students. Our results suggest that the Internet activities of Polish high school students are only weakly associated with how they handle health issues.

### Limitations

The survey reported in our paper was performed on high school students from only one voivodship. Therefore, the observations described here may not represent the whole population of high school students in Poland.

It is also not fully clear if the version of the HLS-EU-Q47 questionnaire developed for the general population is appropriate for surveys conducted on adolescents [6]. Although this tool was developed and validated for a population aged 15 and older, some authors tend to question the ability of adolescents to understand some terms used in the HLS-EU-Q47. The analysis carried out by Domanska et al. showed that adolescents aged 14–17 were unfamiliar with some terms used in the questionnaire or provided heterogeneous interpretations of the terms [46]. Furthermore, they had limited or no experience of some health-related tasks regarding healthcare and disease prevention included on the questionnaire. However, it should also be noted that the survey among Polish students was addressed to a more mature youth population with a mean age of 17 years.

Finally, we must admit that in our analysis we have covered only a portion of the possible determinants of adolescent health literacy. It is clear, both from Manganello’s framework [40] and the 6-D model of HL in childhood and youth proposed by Bröder et al. [44] that many factors play a role in determining the level of health literacy in the target group. Our study was mainly focused on, following Manganello’s nomenclature for individual traits, family influences and education system features.

## 5. Conclusions

The analysis reported in this paper confirmed that the prevalence of limited HL is greater in respondents whose parents have lower levels of education, who live in households with a smaller inhabitable area, are not receiving external support, have worse self-assessment of their economic situation, spend the smallest sums for a mobile phone and have at home the smallest collection of books. HL sub-indices for the seven main areas, including accessing, understanding, appraising and the use of health information, healthcare, disease prevention and health promotion, are consistently associated with the mothers’ level of education, receiving external support, self-assessed economic situation, the level of expenditure on mobile phones and the size of the home’s collection of books.

The student’s gender and the level of education achieved by their father were associated only with selected sub-indices, similarly with the inhabitable area of a household. The type of school and the class attended at the time of the survey were associated only with individual sub-indices. No associations with HL sub-indices were found for the marital status of their parents, the number of people in the household and, surprisingly, with the intensity of Internet use.

## Figures and Tables

**Table 1:** Cronbach-α and Guttman split-half coefficients for HL indices used in the analysis.

HL Index	Cronbach-α Coefficient	Guttman Split-Half Coefficient
AI-HL	0.831	0.750
UI-HL	0.812	0.762
ApI-HL	0.851	0.802
UsI-HL	0.814	0.690
HC-HL	0.856	0.790
DP-HL	0.875	0.752
HP-HL	0.889	0.824
General HL	0.950	0.882

**Table 2:** Characteristics of the study group.

Variable	Categories	%	*n*
Gender	female	66.3	1457
	male	33.7	741
Class at school	I class	37.0	809
	II class	28.8	630
	III or IV class	34.2	748
Type of school	general education	82.3	1829
	providing vocational training	17.7	393
Education level of mother	primary or vocational	24.5	540
	secondary	37.4	825
	University education	38.2	844
Education level of father	primary or vocational	40.1	540
	secondary	33.1	825
	University education	26.8	844
Marital status of parents	married	86.6	1918
	divorced or in separation	10.2	227
	one or both parents	3.2	71
Number of household members	<3 household members	4.2	93
	3	16.7	370
	4	33.3	734
	5	22.1	487
	6	13.3	294
	7	5.7	126
	>7	4.7	103
Place of residence	rural	51.5	1142
	urban ≤ 10,000	6.0	132
	urban > 10,000 to 200,000	19.3	427
	urban > 200,000	23.2	515
Size of accommodation	≤50 m^2^	9.7	213
	>50 m^2^–70 m^2^	15.4	339
	>70 m^2^–90 m^2^	11.6	255
	>90 m^2^	63.3	1393
Monthly expenses on mobile phone	<5 PLN	5.1	112
	5–<10 PLN	6.6	144
	10–<30 PLN	32.6	716
	30–<50 PLN	34.0	747
	≥50 PLN	21.7	475
Receiving external support	no	45.4	972
	yes	54.6	1170
Self-assessed economic situation	very bad, bad or average	13.8	305
	good	53.4	1177
	very good	32.8	722
Number of books at home	≤25	12.4	272
	26–50	17.9	393
	51–100	27.5	605
	101–500	32.7	719
	>500	9.5	209
Weekly duration of Internet use	≥2 h	9.1	202
	>2–7 h	20.4	454
	>7–14 h	18.3	405
	>14–21 h	16.3	361
	>21–35 h	16.0	354
	>35 h	19.9	440
Health literacy	inadequate	4.6	96
	problematic	36.8	766
	sufficient	45.2	942
	excellent	13.4	278

**Table 3:** Univariate and multivariate logistic regression models developed for the prevalence of limited HL.

Variable	Categories	OR	95% CI	*p*	aOR	a95% CI	*p*
Gender	female *						
	male	0.87	0.72–1.05	0.143			
Class at school	I class *						
	II class	1.03	0.82–1.28	0.818			
	III or IV class	1.22	0.99–1.51	0.059			
Type of school	GE *						
	with VT	1.15	0.92–1.45	0.216			
Education level of mother ^#^	primary or vocational *						
	secondary	0.97	0.78–1.22	0.821	0.93	0.72–1.21	0.934
	University	0.73	0.58–0.92	0.007	0.74	0.55–0.98	0.038
Education level of father ^#^	primary or vocational *						
	secondary	1.10	0.89–1.35	0.381	1.32	1.04–1.67	0.022
	University	0.80	0.64–0.99	0.047	1.12	0.84–1.50	0.424
Marital status of parents	married *						
	divorced or separated	1.10	0.83–1.46	0.514			
	one or both parents	1.04	0.64–1.70	0.865			
Number of household members	<3 household members *						
	3	1.20	0.74–1.94	0.461			
	4	1.10	0.70–1.74	0.684			
	5	1.18	0.73–1.89	0.497			
	6	1.21	0.74–1.98	0.452			
	7	1.16	0.65–2.04	0.620			
	>7	1.35	0.74–2.44	0.326			
Place of residence	rural *						
	urban ≤ 10,000	0.96	0.65–1.40	0.826			
	urban > 10,000 to 200,000	0.97	0.76–1.22	0.767			
	urban > 200,000	1.06	0.85–1.31	0.622			
Size of accommodation ^#^	≤50 m^2^ *						
	>50 m^2^–70 m^2^	0.93	0.65–1.33	0.686	1.08	0.73–1.58	0.714
	>70 m^2^–90 m^2^	0.58	0.39–0.85	0.005	0.66	0.43–0.998	0.049
	>90 m^2^	0.74	0.54–0.99	0.045	0.92	0.66–1.28	0.920
Monthly expenses on mobile phone ^#^	≤5 PLN *						
	>5–10 PLN	0.63	0.37–1.06	0.082	0.64	0.37–1.12	0.116
	>10–30 PLN	0.80	0.52–1.21	0.286	0.85	0.55–1.33	0.481
	>30–50 PLN	0.79	0.52–1.20	0.277	0.89	0.58–1.39	0.612
	>50 PLN	0.62	0.40–0.95	0.030	0.70	0.44–1.11	0.126
Receiving external support ^#^	no *						
	yes	0.81	0.68–0.97	0.021	0.88	0.73–1.07	0.198
Self-assessed economic situation ^#^	worse than good *						
	good	0.59	0.46–0.77	0.000	0.65	0.49–0.86	0.003
	very good	0.37	0.28–0.49	0.000	0.43	0.32–0.59	<0.001
Number of books at home ^#^	≤25 *						
	26–50	1.24	0.89–1.72	0.196	1.19	0.84–1.68	0.331
	51–100	1.03	0.76–1.39	0.870	1.03	0.75–1.42	0.861
	101–500	1.03	0.76–1.38	0.866	1.11	0.80–1.53	0.530
	>500	0.66	0.44–0.98	0.038	0.79	0.52–1.21	0.278
Weekly duration of Internet use	not more than 2 h *						
	>2–7 h	1.12	0.79–1.59	0.518			
	>7–14	1.23	0.86–1.75	0.259			
	>14–21	1.18	0.82–1.69	0.381			
	>21–35	0.90	0.62–1.30	0.566			
	>35 h	1.22	0.86–1.74	0.259			

*—referential categories in logistic regression models; ^#^—variables included in the multivariate logistic regression model; *p*—*p* values for univariate and multivariate logistic regression models, OR—odds ratio, 95% CI—95% confidential interval; aOR—adjusted odds ratio, a95% CI—adjusted 95% CI.

**Table 4:** Univariate linear regression models for AI-HL and UI-HL.

Variable	Categories	AI-HL		UI-HL	
		B(SE)	*p*	B(SE)	*p*
Gender	male vs. female	0.96 (0.32)	0.002	−0.10 (0.32)	0.763
Class at school	I class *				
	II class	0.28 (0.38)	0.449	0.7 (0.37)	0.062
	III or IV class	−0.54 (0.36)	0.125	0.14 (0.36)	0.690
Type of school	with VT vs. GE	−0.30 (0.39)	0.437	−1.18 (0.39)	0.002
Education level of mother	secondary *				
	primary or voc.	0.16 (0.39)	0.682	0.11 (0.39)	0.785
	University	1.15 (0.34)	0.001	1.13 (0.34)	0.001
Education level of father	primary or voc. *				
	secondary	−0.03 (0.35)	0.939	−0.64 (0.35)	0.070
	University	0.88 (0.37)	0.019	0.91 (0.37)	0.014
Marital status of parents	married *				
	div. or in sep.	−0.79 (0.49)	0.107	−0.14 (0.49)	0.775
	one or both parents deceased	−0.35 (0.83)	0.672	0.39 (0.84)	0.641
Number of household members	4 *				
	<3	0.12 (0.79)	0.876	0.38 (0.77)	0.625
	3	−0.11 (0.45)	0.805	−0.12 (0.44)	0.788
	5	−0.35 (0.41)	0.399	−0.26 (0.41)	0.521
	6	−0.33 (0.49)	0.502	0.01 (0.49)	0.992
	7	−0.37 (0.68)	0.585	−0.69 (0.68)	0.314
	>7	0.09 (0.73)	0.898	−0.08 (0.76)	0.920
Place of residence	rural *				
	urban ≤ 10,000	0.47 (0.65)	0.467	0.33 (0.64)	0.607
	urban > 10,000 to 200,000	−0.07 (0.40)	0.869	0.17 (0.40)	0.671
	urban >200,000	−0.37 (0.37)	0.325	0.49 (0.37)	0.186
Size of accommodation	≤50 m^2^ *				
	>50 m^2^–70 m^2^	−0.86 (0.61)	0.159	0.01 (0.61)	0.989
	>70 m^2^–90 m^2^	0.96 (0.58)	0.097	0.7 (0.58)	0.226
	>90 m^2^	0.96 (0.42)	0.021	0.35 (0.42)	0.405
Monthly expenses on mobile phone	30–<50 PLN *				
	<5 PLN	−1.49 (0.74)	0.044	−1.47 (0.71)	0.039
	5–<10 PLN	0.64 (0.64)	0.320	0.37 (0.63)	0.556
	10–<30 PLN	0.05 (0.37)	0.889	−0.13 (0.37)	0.733
	from 50 PLN	1 (0.41)	0.014	0.07 (0.41)	0.868
Receiving external support	yes vs. no	0.28 (0.3)	0.358	0.6 (0.3)	0.050
Self-assessed economic situation	good				
	worse than good	−1.44 (0.44)	0.001	−0.38 (0.45)	0.400
	very good	2.01 (0.33)	<0.001	1.36 (0.33)	<0.001
Number of books at home	101–500 *				
	≤25	0.16 (0.50)	0.751	−0.53 (0.5)	0.295
	26–50	−0.75 (0.44)	0.089	−0.79 (0.44)	0.071
	51–100	−0.01 (0.39)	0.984	−0.29 (0.38)	0.457
	>500	0.93 (0.55)	0.088	1.57 (0.55)	0.004
Weekly duration of Internet use	>2–7 h *				
	≤2 h	0.61 (0.59)	0.300	0.51 (0.59)	0.385
	>7–14	−0.36 (0.48)	0.453	−0.52 (0.48)	0.281
	>14–21	−0.04 (0.50)	0.943	−0.16 (0.5)	0.752
	>21–35	0.77 (0.50)	0.120	0.15 (0.5)	0.768
	>35 h	0.37 (0.47)	0.429	−0.13 (0.47)	0.790

Abbreviations: *—referential category of variable, B (SE)—unstandardised regression coefficient (standard error), *p*—*p*-value for a univariate linear regression model, VT—vocational training, GE—general education, div.—divorced, voc.—vocational.

**Table 5:** Univariate linear regression models for ApI-HL and UsI-HL.

Variable	Categories	ApI-HL		UsI-HL	
		B(SE)	*p*	B(SE)	*p*
Gender	male vs. female	0.7 (0.35)	0.045	0.22 (0.33)	0.495
Class at school	I class *				
	II class	0.28 (0.41)	0.500	−0.04 (0.39)	0.912
	III or IV class	0.05 (0.39)	0.895	−0.67 (0.37)	0.067
Type of school	with VT vs. GE	−0.20 (0.43)	0.645	−0.05 (0.4)	0.904
Education level of mother	secondary *				
	primary or voc.	−0.17 (0.43)	0.692	0.08 (0.40)	0.833
	University	1.19 (0.38)	0.002	0.99 (0.35)	0.005
Education level of father	primary or voc. *				
	secondary	−0.72 (0.39)	0.066	−0.21 (0.36)	0.573
	University	0.70 (0.41)	0.090	0.31 (0.39)	0.426
Marital status of parents	married *				
	div or in sep.	−0.06 (0.54)	0.906	−0.48 (0.5)	0.340
	one or both parents deceased	−0.13 (0.93)	0.890	−0.09 (0.87)	0.920
Number of household members	4 *				
	<3	0.95 (0.84)	0.259	0 (0.79)	0.999
	3	0.29 (0.49)	0.557	0.05 (0.46)	0.921
	5	−0.22 (0.45)	0.623	−0.51 (0.42)	0.226
	6	−0.19 (0.54)	0.723	0.03 (0.50)	0.952
	7	−0.55 (0.75)	0.463	−0.17 (0.71)	0.814
	>7	0.44 (0.83)	0.598	−0.23 (0.78)	0.768
Place of residence	rural				
	urban ≤ 10,000	0.35 (0.72)	0.625	−0.35 (0.67)	0.598
	urban > 10,000 to 200,000	−0.30 (0.44)	0.495	−0.05 (0.41)	0.909
	urban > 200,000	−0.11 (0.41)	0.797	−0.06 (0.38)	0.871
Size of accommodation	≤50 m^2^ *				
	>50 m^2^–70 m^2^	−0.09 (0.68)	0.894	0.23 (0.64)	0.713
	>70 m^2^–90 m^2^	1.25 (0.64)	0.052	0.91 (0.6)	0.127
	>90 m^2^	0.67 (0.47)	0.151	0.93 (0.44)	0.033
Monthly expenses on mobile phone	30–<50 PLN *				
	<5 PLN	−0.89 (0.79)	0.262	−2.11 (0.75)	0.005
	5–<10 PLN	0.39 (0.7)	0.578	−0.12 (0.66)	0.855
	10–<30 PLN	−0.24 (0.4)	0.545	−0.28 (0.38)	0.458
	from 50 PLN	1.04 (0.45)	0.021	0.81 (0.42)	0.056
Receiving external support	yes vs. no	0.77 (0.34)	0.023	0.54 (0.32)	0.086
Self-assessed economic situation	good *				
	worse than good	−1.35 (0.49)	0.006	−1.28 (0.46)	0.005
	very good	2.25 (0.36)	<0.001	2.24 (0.34)	<0.001
Number of books at home	101–500 *				
	≤25	0.27 (0.55)	0.625	0.31 (0.52)	0.551
	26–50	−0.82 (0.48)	0.089	−0.23 (0.45)	0.612
	51–100	−0.03 (0.43)	0.939	<0.001 (0.40)	0.999
	>500	2.26 (0.61)	<0.001	1.48 (0.57)	0.010
Weekly duration of Internet use	>2–7 h *				
	≤2 h	0.39 (0.66)	0.552	−0.2 (0.61)	0.745
	>7–14	−0.56 (0.53)	0.285	−0.5 (0.49)	0.311
	>14–21	−0.57 (0.55)	0.294	−0.62 (0.51)	0.226
	>21–35	0.51 (0.55)	0.354	−0.33 (0.51)	0.517
	>35 h	0.12 (0.52)	0.813	−0.16 (0.49)	0.740

Abbreviations: *—referential category of variable, B (SE)—unstandardised regression coefficient (standard error), *p*—*p*-value for a univariate linear regression model, VT—vocational training, GE—general education, div.—divorced, voc.—vocational.

**Table 6:** Univariate linear regression models for HL sub-indices for appraisal and use of health information.

Variable	Categories	HC-HL		DP-HL		HP-HL	
		B(SE)	*p*	B(SE)	*p*	B(SE)	*p*
Gender	male vs. female	0.07 (0.29)	0.818	0.62 (0.35)	0.073	0.68 (0.35)	0.051
Class at school	I class *						
	II class	0.36 (0.34)	0.290	0.18 (0.41)	0.661	0.11 (0.41)	0.786
	III or IV class	−0.03 (0.32)	0.923	0.09 (0.39)	0.817	−0.93 (0.39)	0.017
Type of school	with VT vs. GE	−0.54 (0.35)	0.123	−0.67 (0.42)	0.112	0.04 (0.43)	0.924
Education level of mother	secondary *						
	primary or voc.	0.12 (0.35)	0.733	0.08 (0.43)	0.856	0.03 (0.43)	0.949
	University	0.65 (0.31)	0.035	1.53 (0.37)	<0.001	1.19 (0.38)	0.002
Education level of father	primary or voc. *						
	secondary	−0.4 (0.32)	0.211	−0.52 (0.38)	0.179	−0.34 (0.39)	0.374
	University	0.64 (0.34)	0.057	1.29 (0.41)	0.002	0.35 (0.41)	0.401
Marital status of parents	married *						
	div or in sep.	−0.23 (0.44)	0.592	−0.85 (0.53)	0.109	0.2 (0.53)	0.706
	one or both parents deceased	−0.53 (0.76)	0.483	−0.19 (0.96)	0.842	0.95 (0.93)	0.307
Number of household members	4 *						
	<3	0.25 (0.7)	0.718	0.8 (0.86)	0.352	0.75 (0.86)	0.383
	3	−0.12 (0.4)	0.769	0.08 (0.49)	0.874	0.17 (0.49)	0.727
	5	−0.54 (0.37)	0.146	−0.08 (0.45)	0.862	−0.12 (0.45)	0.793
	6	0.08 (0.44)	0.861	−0.1 (0.53)	0.845	−0.23 (0.54)	0.675
	7	−0.53 (0.61)	0.384	−0.14 (0.75)	0.854	−0.03 (0.75)	0.968
	>7	−0.13 (0.69)	0.850	0.18 (0.81)	0.826	0.60 (0.83)	0.465
Place of residence	rural *						
	urban ≤ 10,000	0.05 (0.59)	0.933	0.37 (0.71)	0.606	0.2 (0.71)	0.780
	urban > 10,000 to 200,000	0.07 (0.36)	0.851	−0.03 (0.44)	0.940	−0.25 (0.44)	0.561
	urban >200,000	0.12 (0.33)	0.730	−0.03 (0.41)	0.939	−0.25 (0.41)	0.535
Size of accommodation	≤50 m^2^ *						
	>50 m^2^–70 m^2^	0.72 (0.55)	0.193	−1.17 (0.67)	0.08	−0.56 (0.68)	0.408
	>70 m^2^–90 m^2^	1.47 (0.52)	0.005	0.37 (0.63)	0.554	0.70 (0.63)	0.268
	>90 m^2^	0.92 (0.38)	0.015	0.39 (0.46)	0.407	0.56 (0.46)	0.226
Monthly expenses on mobile phone	30–<50 PLN *						
	<5 PLN	−1.02 (0.65)	0.115	−1.24 (0.8)	0.121	−2.41 (0.79)	0.002
	5–<10 PLN	0.53 (0.58)	0.357	0.79 (0.7)	0.263	0.1 (0.70)	0.883
	10–<30 PLN	−0.41 (0.33)	0.218	0.19 (0.4)	0.646	−0.36 (0.40)	0.365
	from 50 PLN	0.76 (0.37)	0.040	0.69 (0.45)	0.124	0.69 (0.45)	0.124
Receiving external support	yes vs. no	0.68 (0.27)	0.014	0.52 (0.33)	0.118	0.31 (0.33)	0.360
Self-assessed economic situation	good *						
	worse than good	−0.56 (0.4)	0.163	−1.27 (0.48)	0.009	−1.77 (0.48)	<0.001
	very good	1.87 (0.29)	<0.001	1.95 (0.36)	<0.001	1.89 (0.36)	<0.001
Number of books at home	101–500 *						
	≤25	−0.59 (0.45)	0.187	−0.20 (0.55)	0.714	0.80 (0.55)	0.147
	26–50	−0.68 (0.40)	0.088	−1.10 (0.48)	0.021	−0.45 (0.48)	0.349
	51–100	−0.21 (0.35)	0.547	−0.46 (0.42)	0.282	0.47 (0.42)	0.267
	>500	1.47 (0.50)	0.003	1.60 (0.60)	0.007	1.51 (0.6)	0.012
Weekly duration of Internet use	>2–7 h *						
	≤2 h	0.48 (0.53)	0.373	0.14 (0.65)	0.827	0.80 (0.65)	0.216
	>7–14	−0.47 (0.43)	0.278	0.06 (0.51)	0.900	−0.64 (0.53)	0.226
	>14–21	−0.13 (0.45)	0.771	−0.54 (0.53)	0.309	−0.69 (0.54)	0.205
	>21–35	0.24 (0.45)	0.591	−0.28 (0.55)	0.608	0.03 (0.54)	0.952
	>35 h	0.13 (0.42)	0.763	0.58 (0.55)	0.291	0.20 (0.52)	0.703

Abbreviations: *—referential category of variable, B (SE)—unstandardised regression coefficient (standard error), *p*—*p*-value for a univariate linear regression model, VT—vocational training, GE—general education, div.—divorced, voc.—vocational.

## Data Availability

The data that support the findings of this study are available on request from the corresponding author. The data are not publicly available due to privacy and ethical restrictions. The authors did not include in the information about the study provided to the participants that the public access to the data obtained during the survey may be considered. Access to the data will be granted on a case-by-case basis on a justified request after receiving consent from the Bioethical Committee at Jagiellonian University.

## Supplementary Materials

The following are available online at https://www.mdpi.com/article/10.3390/ijerph182212231/s1, Supplementary File S1 includes the items of the questionnaire applied in the survey used as a source of independent variables in regression modelling.

## References

[1] Nutbeam D., Kickbusch I. (1998). Health Promotion Glossary. Health Promot. Int..

[2] Zaben K., Khalil A. (2019). Health Literacy, Self-Care Behavior and Quality of Life in Acute Coronary Syndrome Patients: An Integrative Review. Open J. Nurs..

[3] Duplaga M. (2020). Determinants and Consequences of Limited Health Literacy in Polish Society. Int. J. Environ. Res. Public Health.

[4] Berkman N.D., Sheridan S.L., Donahue K.E., Halpern D.J., Crotty K. (2011). Low Health Literacy and Health Outcomes: An Updated Systematic Review. Ann. Intern. Med..

[5] Humphrys E., Burt J., Rubin G., Emery J.D., Walter F.M. (2019). The Influence of Health Literacy on the Timely Diagnosis of Symptomatic Cancer: A Systematic Review. Eur. J. Cancer Care.

[6] Sørensen K., Pelikan J.J.M., Röthlin F., Ganahl K., Slonska Z., Doyle G., Fullam J., Kondilis B., Agrafiotis D., Uiters E. (2015). Health Literacy in Europe: Comparative Results of the European Health Literacy Survey (HLS-EU). Eur. J. Public Health.

[7] World Health Organization (2017). Shanghai Declaration on Promoting Health in the 2030 Agenda for Sustainable Development. Health Promot. Int..

[8] Olives T., Patel R., Patel S., Hottinger J., Miner J.R. (2011). Health Literacy of Adults Presenting to an Urban ED. Am. J. Emerg. Med..

[9] Rikard R.V., Thompson M.S., McKinney J., Beauchamp A. (2016). Examining Health Literacy Disparities in the United States: A Third Look at the National Assessment of Adult Literacy (NAAL). BMC Public Health.

[10] Toçi E., Burazeri G., Myftiu S., Sørensen K., Brand H. (2016). Health Literacy in a Population-Based Sample of Adult Men and Women in a South Eastern European Country. J. Public Health.

[11] Ozkan S., Dikmen A., Tuzun H., Karakaya K. (2016). Prevalence and Determiners of Health Literacy in Turkey. Eur. J. Public Health.

[12] Matsumoto S., Takamura H., Okumura M. (2005). Sentiment Classification Using Word Sub-Sequences and Dependency Sub-Trees. Pacific-Asia Conference on Knowledge Discovery and Data Mining.

[13] Duong T.V., Aringazina A., Baisunova G., Nurjanah, Pham T.V., Pham K.M., Truong T.Q., Nguyen K.T., Oo W.M., Mohamad E. (2017). Measuring Health Literacy in Asia: Validation of the HLS-EU-Q47 Survey Tool in Six Asian Countries. J. Epidemiol..

[14] Altin S.V., Finke I., Kautz-Freimuth S., Stock S. (2014). The Evolution of Health Literacy Assessment Tools: A Systematic Review. BMC Public Health.

[15] Okan O., Lopes E., Bollweg T.M., Bröder J., Messer M., Bruland D., Bond E., Carvalho G.S., Sørensen K., Saboga-Nunes L. (2018). Generic Health Literacy Measurement Instruments for Children and Adolescents: A Systematic Review of the Literature. BMC Public Health.

[16] Marques S.R.L., Lemos S.M.A. (2017). Health Literacy Assessment Instruments: Literature Review. Audiol. Commun. Res..

[17] Liu H., Zeng H., Shen Y., Zhang F., Sharma M., Lai W., Zhao Y., Tao G., Yuan J., Zhao Y. (2018). Assessment Tools for Health Literacy among the General Population: A Systematic Review. Int. J. Environ. Res. Public Health.

[18] Perry E.L. (2014). Health Literacy in Adolescents: An Integrative Review. J. Spec. Pediatr. Nurs..

[19] Davis T.C., Wolf M.S., Arnold C.L., Byrd R.S., Long S.W., Springer T., Estela K., Bocchini J.A. (2006). Development and Validation of the Rapid Estimate of Adolescent Literacy in Medicine (REALM-Teen): A Tool to Screen Adolescents for below-Grade Reading in Health Care Settings. Pediatrics.

[20] Vardavas C.I., Kondilis B.K., Patelarou E., Akrivos P.D., Falagas M.E. (2009). Health Literacy and Sources of Health Education among Adolescents in Greece. Int. J. Adolesc. Med. Health.

[21] Röthlin F., Pelikan J., Ganahl K. (2013). Die Gesundheitskompetenz von 15-Jährigen Jugendlichen in Österreich.

[22] Driessnack M., Chung S., Perkhounkova E., Hein M. (2014). Using the “Newest Vital Sign” to Assess Health Literacy in Children. J. Pediatr. Health Care.

[23] Bröder J., Okan O., Bauer U., Bruland D., Schlupp S., Bollweg T.M., Saboga-Nunes L., Bond E., Sørensen K., Bitzer E.M. (2017). Health Literacy in Childhood and Youth: A Systematic Review of Definitions and Models. BMC Public Health.

[24] Wu A.D., Begoray D.L., MacDonald M., Higgins J.W., Frankish J., Kwan B., Fung W., Rootman I. (2021). Developing and Evaluating a Relevant and Feasible Instrument for Measuring Health Literacy of Canadian High School Students. Health Promot. Int..

[25] Dehghankar L., Panahi R., Kekefallah L., Hosseini N., Hasannia E. (2019). The Study of Health Literacy and Its Related Factors among Female Students at High Schools in Qazvin. J. Health Lit..

[26] Lam L.T., Yang L. (2014). Is Low Health Literacy Associated with Overweight and Obesity in Adolescents: An Epidemiology Study in a 12–16 Years Old Population, Nanning, China, 2012. Arch. Public Health.

[27] Park A., Eckert T.L., Zaso M.J., Scott-Sheldon L.A.J., Vanable P.A., Carey K.B., Ewart C.K., Carey M.P. (2017). Associations between Health Literacy and Health Behaviors among Urban High Schoolers. J. Sch. Health.

[28] Sansom-Daly U.M., Lin M., Robertson E.G., Wakefield C.E., McGill B.C., Girgis A., Cohn R.J. (2016). Health Literacy in Adolescents and Young Adults: An Updated Review. J. Adolesc. Young Adult Oncol..

[29] Fleary S.A., Joseph P., Pappagianopoulos J.E. (2018). Adolescent Health Literacy and Health Behaviors: A Systematic Review. J. Adolesc..

[30] Wallston K.A., Wallston B.S., DeVellis R. (1978). Development of the Multidimensional Health Locus of Control (MHLC) Scales. Health Educ. Behav..

[31] Pelikan J.M., Röthlin F., Ganahl K. Measuring Comprehensive Health Literacy in General Populations: Validation of Instrument, Indices and Scales of the HLS-EU Study. Proceedings of the 6th Annual Health Literacy Research Conference.

[32] Reid A.L., Porter K.J., You W., Kirkpatrick B.M., Yuhas M., Vaught S.S., Zoellner J.M. (2021). Low Health Literacy Is Associated With Energy-Balance-Related Behaviors, Quality of Life, and BMI Among Rural Appalachian Middle School Students: A Cross-Sectional Study. J. Sch. Health.

[33] Chi X., Liang K., Chen S.T., Huang Q., Huang L., Yu Q., Jiao C., Guo T., Stubbs B., Hossain M.M. (2021). Mental Health Problems among Chinese Adolescents during the COVID-19: The Importance of Nutrition and Physical Activity. Int. J. Clin. Health Psychol..

[34] Wright L.J., Williams S.E., van Zanten J.J.C.S.V. (2021). Physical Activity Protects Against the Negative Impact of Coronavirus Fear on Adolescent Mental Health and Well-Being During the COVID-19 Pandemic. Front. Psychol..

[35] Ruíz-Roso M.B., de Carvalho Padilha P., Matilla-Escalante D.C., Brun P., Ulloa N., Acevedo-Correa D., Ferreira Peres W.A., Martorell M., Bousquet Carrilho T.R., De Oliveira Cardoso L. (2020). Changes of Physical Activity and Ultra-Processed Food Consumption in Adolescents from Different Countries during Covid-19 Pandemic: An Observational Study. Nutrients.

[36] Zenic N., Taiar R., Gilic B., Blazevic M., Maric D., Pojskic H., Sekulic D. (2020). Levels and Changes of Physical Activity in Adolescents during the COVID-19 Pandemic: Contextualizing Urban vs. Rural Living Environment. Appl. Sci..

[37] Bates L.C., Zieff G., Stanford K., Moore J.B., Kerr Z.Y., Hanson E.D., Gibbs B.B., Kline C.E., Stoner L. (2020). COVID-19 Impact on Behaviors across the 24-Hour Day in Children and Adolescents: Physical Activity, Sedentary Behavior, and Sleep. Children.

[38] Rossi L., Behme N., Breuer C. (2021). Physical Activity of Children and Adolescents during the COVID-19 Pandemic—A Scoping Review. Int. J. Environ. Res. Public Health.

[39] Sekulic D., Ostojic D., Decelis A., Castro-Piñero J., Jezdimirovic T., Drid P., Ostojic L., Gilic B. (2021). The Impact of Scholastic Factors on Physical Activity Levels during the COVID-19 Lockdown: A Prospective Study on Adolescents from Bosnia and Herzegovina. Children.

[40] Manganello J.A. (2008). Health Literacy and Adolescents: A Framework and Agenda for Future Research. Health Educ. Res..

[41] Nielsen-Bohlman L., Panzer A., Kindig D., Nielsen-Bohlman L., Panzer A.M., Kindig D.A. (2004). Health Literacy: A Prescription to End Confusion.

[42] Bronfenbrenner U. (1979). The Ecology of Human Development: Experiments by Nature and Design.

[43] Guo S., Naccarella L., Yu X., Armstrong R., Browne G., Shi Y., Davis E. (2021). Health Literacy and Its Mediating Role in Predicting Health Behaviors Among Chinese Secondary Students. Asia-Pac. J. Public Health.

[44] Bröder J., Okan O., Bauer U., Bollweg T.M., Bruland D., Pinheiro P. (2019). Child and Youth Health Literacy: A Conceptual Analysis and Proposed Target-Group-Centred Definition. Int. J. Environ. Res. Public Health.

[45] Thomas Y., Srinivasan R. (2016). Emerging Shifts in Learning Paradigms—From Millenials to the Digital Natives. Int. J. Appl. Eng. Res..

[46] Domanska O.M., Firnges C., Bollweg T.M., Sørensen K., Holmberg C., Jordan S. (2018). Do Adolescents Understand the Items of the European Health Literacy Survey Questionnaire (HLS-EU-Q47)—German Version? Findings from Cognitive Interviews of the Project “Measurement of Health Literacy Among Adolescents” (MOHLAA) in Germany. Arch. Public Health.

