# Supplementary material for: Socio-Economic Determinants of Health Literacy in High School Students: A Cross-Sectional Study
Source: Int J Environ Res Public Health. 2021 Nov 21;18(22):12231. doi: 10.3390/ijerph182212231 (PMC8624924; doi:10.3390/ijerph182212231)
Supplement: Supplementary file 1 [file ijerph-18-12231-s001.zip › ijerph-1424720-supplementary.pdf]

Questionnaire's items operationalised in the analysis

Gender □ female □ male

Class at school ______________ (1, 2, 3 or 4?)

Type of the school

□ lyceum

□ technical vocational school

Educational level of mother (legal guardian)

□ elementary

□ basic vocationally

□ secondary

□ university

Educational level of father (legal guardian)

□ elementary

□ basic vocationally

□ secondary

□ university

What is a marital status of your parents

□ marriage

□ concubine

□ divorced or in separation

□ one of parents died

□ both parents died

What is the number of household members?

how many persons?_____________________

Place of residence

□ urban >400,000 inhabitants

□ urban >200,000 to 400,000 inhabitants

□ urban >100,000 to 200,000 inhabitants

□ urban >10,000 to 100,000 inhabitants

□ urban ≤10,0000 inhabitants

□ rural

What is the size of your accommodation?

□ not more than 30 m^2

□ from 31 m^2 to 50 m^2

□ from 51 m^2 to 70 m^2

□ from 71 m^2 to 90 m^2

□ above 90 m^2

How much do you spend monthly on your mobile phone?

□ below 5 zł

□ from 5 to below 10 zł

□ from 10 to below 30 zł

□ from 30 to below 50 zł

□ at least 50 zł

□ I have not got a mobile phone

Does your household receive external help? What is the form of such help?

□ financial

□ material

□ in the form of services

□ no external help

How do you assess economic situation of your family

□ very good

□ good

□ average

□ bad

□ very bad

How many books, not including school manual and e-books, are there at your home?

□ no books

□ not more than 25

□ 26 – 50

□ 51 – 100

□ 101 – 500

□ more than 500

How many hours per week do you use the Internet?

□ ≤2 hours

□ 3 - 7 hours

□ 8 - 14 hours

□ 15 - 21 hours

□ 22- 35 hours

18. Jakie jest źródło utrzymania rodziny?
(zaznacz wszystkie możliwe)

□ pracują obydwoje rodzice (opiekunowie)

□ pracuje jeden z rodziców (opiekunów), kto z rodziców? ________________

□ obydwoje rodzice (opiekunowie) są bezrobotni

□ renta/emerytura, kto z rodziców?________________

□ prowadzenie gospodarstwa rolnego

□ prowadzenie działalności gospodarczej

□ praca dorywcza, kto z rodziców?________________

□ praca za granicą, kto z rodziców?________________

19.Czy gospodarstwo domowe korzysta z pomocy z zewnątrz? Jaka jest jej forma? (np. od pomocy społecznej, zasiłki dla bezrobotnych itd.)

□ finansowa

□ rzeczowa

□ w formie usług

□ gospodarstwo domowe nie uzyskuje pomocy z zewnątrz

20. Jak oceniasz sytuację materialną/finansową w rodzinie?

□ bardzo dobra

□ dobra

□ średnia

□ zła

□ bardzo zła

21. Jakie dobra są w posiadaniu Twojej rodziny?
(zaznacz wszystkie możliwe)

□ samochód osobowy, osobowo-dostawczy, ile?________

□ iPad lub inny tablet, ile?__________________________

□ komputer stacjonarny, laptop, notebooki, ile?________

□ dostęp do Internetu w domu

□ telewizja kablowa / satelitarna

□ telefon komórkowy, ile?__________________________

22.Jakie urządzenia elektroniczne posiadasz na własny użytek? (zaznacz wszystkie możliwe)

□ telefon komórkowy

□ iPad lub inny tablet

□ komputer stacjonarny, laptop, notebook

□ telewizor

□ elektroniczny czytnik książek (e-book)

23.Jaką kwotę przeznaczasz miesięcznie na własny telefon komórkowy?

□ do 5 zł

□ 5 – 10 zł

□ 10 – 30 zł

□ 30 – 50 zł

□ powyżej 50 zł

□ nie posiadam telefonu komórkowego

24. Ile książek (bez podręczników szkolnych i e-booków) jest w Twoim domu?

□ brak zbiorów książek

□ do 25 sztuk

□ 26 – 50 sztuk

□ 51 – 100 sztuk

□ 101 – 500 sztuk

□ ponad 500 sztuk

□ powyżej 36 godzin
